# Chemical Characterization and Protective Effects of a Subcritical Water Extract from Olive Pomace Against Dyslipidemia and Hepatic Steatosis in High-Fat/High-Sugar Diet–Fed Mice

**DOI:** 10.3390/molecules31060995

**Published:** 2026-03-16

**Authors:** Alicia Ochoa-Acosta, Analy Aispuro-Pérez, Feliznando Cárdenas-Torres, Mayra Arias-Gastelum, Marco Antonio Valdez-Flores, María de la Paz Espinoza, Julio Montes-Avila, Bianca Amezquita-López, Roberto Avena-Bustillos, Selina C. Wang, Eli Terán-Cabanillas, Ulises Osuna-Martínez

**Affiliations:** 1 Facultad de Ciencias de la Nutrición y Gastronomía, Universidad Autónoma de Sinaloa, Avenida Cedros y Calle Sauces S/N, Culiacán 80019, Sinaloa, Mexico; daochoa@uas.edu.mx (A.O.-A.); feliznando@uas.edu.mx (F.C.-T.); mayra.arias@uas.edu.mx (M.A.-G.); 2 Facultad de Ciencias Químico Biológicas, Universidad Autónoma de Sinaloa, Ciudad Universitaria, Culiacán 80013, Sinaloa, Mexico; est.analyap@uas.edu.mx (A.A.-P.); jmontes@uas.edu.mx (J.M.-A.); bamezquita@uas.edu.mx (B.A.-L.); 3 Facultad de Medicina, Universidad Autónoma de Sinaloa, Avenida Cedros y Calle Sauces Frac los Fresnos, Culiacán 80019, Sinaloa, Mexico; marco.valdez@uas.edu.mx (M.A.V.-F.); histo.embrio.pato@gmail.com (M.d.l.P.E.); 4 Western Regional Research Center, US Department of Agriculture, Agricultural Research Service, 800 Buchanan Street, Albany, CA 94710, USA; 5 Department of Food Science and Technology, University of California, Davis, CA 95616, USA; scwang@ucdavis.edu

**Keywords:** olive pomace, polyphenols, metabolic syndrome, high-fat and high-sugar diet

## Abstract

Olive pomace, a byproduct of olive oil production, is a rich source of bioactive phenolic compounds with potential health benefits. This study aimed to characterize the chemical composition and evaluate the metabolic effects of a subcritical water extract from California olive pomace (SWE COP) obtained from Arbequina olives. The extract was mainly composed of carbohydrates (72.81%) and contained 66.62 ± 1.22 mg gallic acid equivalents/g of phenolics, with 3,4-DHPEA-EDA, hydroxytyrosol, and verbascoside identified as the predominant compounds. Male C57BL/6N mice were fed a standard diet (SD; n = 7), a high-fat and high-sugar diet (HFSD; n = 7), which was used to induce features of diet-associated metabolic syndrome, or an HFSD supplemented with 3% (*w*/*w*) SWE COP (n = 7) for 16 weeks. Supplementation with SWE COP significantly reduced plasma triglycerides and increased HDL cholesterol levels compared with the HFSD group. Moreover, SWE COP improved glucose tolerance, enhanced insulin sensitivity, and reduced mesenteric and epididymal adiposity. Histological analysis showed that SWE COP alleviated hepatic steatosis and lowered the NAFLD activity score. These findings demonstrate that phenolic-rich SWE COP exerts beneficial effects on glucose and lipid metabolism and reduces liver fat accumulation in diet-induced obese mice. Overall, SWE COP represents a promising functional ingredient derived from olive industry byproducts for mitigating metabolic dysfunctions associated with obesity.

## 1. Introduction

The global rise in metabolic disorders, including obesity, insulin resistance, and non-alcoholic fatty liver disease (NFLD), has become a leading cause of morbidity worldwide [1,2,3,4,5]. This trend is primarily driven by sedentary lifestyles and Western diets rich in saturated fats and simple sugars [6]. These conditions are closely linked to chronic low-grade systemic inflammation and oxidative stress. Specifically, dysfunctional adipose tissue promotes the release of proinflammatory cytokines that impair the signaling and exacerbate dyslipidemia, significantly increasing cardiovascular risk [7,8].

While lifestyle modifications are the frontline treatment, low patient adherence has shifted scientific interest toward sustainable bioactive compounds [9,10]. Polyphenols, particularly those derived from olive trees (*Olea europaea* L.) byproducts, have gained attention due to their potent antioxidant, cardioprotective, and antidiabetic properties [11]. Olive pomace, the primary byproduct of olive extraction, represents 80% of the total waste generated and poses significant environmental challenges [12]. However, it remains a rich source of phenolic compounds such as hydroxytyrosol, oleuropein, and tyrosol, which can improve insulin sensitivity and modulate the lipid profile [13,14,15,16,17].

The efficacy of these extracts is highly dependent on the extraction method. To align with “green chemistry” principles, Subcritical Water Extraction (SWE) has emerged as an ecofriendly alternative to traditional organic solvents [18,19]. By using liquid water at high temperatures and pressures, SWE reduces the dielectric constant of water to levels similar to those of organic solvents, allowing for the efficient recovery of medium-to-low polarity polyphenols while preserving their biological integrity [19,20,21].

Despite the chemical characterization of olive pomace, preclinical studies evaluating the physiological impact of SWE-derived extracts on diet-induced obesity and NAFLD remain limited. Therefore, this study aimed to characterize the phytochemical composition of SWE-extracted olive pomace and evaluate its effects on lipid metabolism and hepatic steatosis in a high-fat/high-sugar diet mouse model.

## 2. Results

### 2.1. SWE COP Chemical Composition

The basic chemical composition of SWE COP is presented in Table 1. Total carbohydrates, calculated by difference, constituted the major component, accounting for up to 72.81% of the extract. This fraction includes soluble and insoluble fiber, as well as other carbohydrates.

### 2.2. Total Soluble Phenolic Content and Individual Phenolic Profile of SWE COP

**Figure 1:**
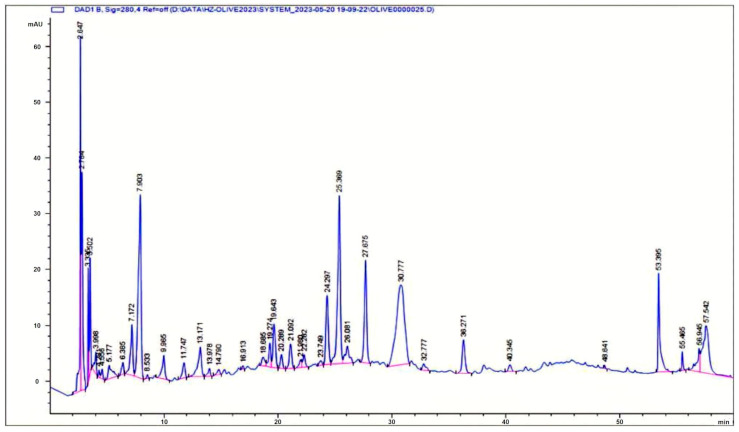
HPLC chromatogram showing identified phenolic compounds in Arbequina SWE COP.

As shown in Table 2, the total extractable phenolic compounds (TPC) of SWE COP were 66.62 ± 1.22 mg acid gallic equivalents per gram of freeze-dried powder olive pomace subcritical water extract. Using HPLC analysis, a total of 18 phenolic compounds were identified and quantified in the Arbequina SWE COP by mass spectrometry with phenolic standards. The corresponding HPLC chromatogram is shown in Figure 1. The identified phenolic compounds included gallic acid, hydroxytyrosol-glucoside, hydroxytyrosol, tyrosol-glucoside, tyrosol, 4-HPA, caffeic acid, vanillin, p-coumaric acid, verbascoside, 3,4 DHPEA-EDA, oleuropein, cinnamic acid, and luteolin. Among these 3,4-DHPEA-EDA (36.6 mg/g of freeze-dried OP SWE) was the predominant compound, followed by hydroxytyrosol (6.5 mg/g of freeze-dried OP SWE) and verbascoside (2.97 mg/g of freeze-dried OP SWE) (Table 2).

### 2.3. Biological Effects of SWE COP

#### 2.3.1. Effects of SWE COP on Food Intake, Energy, Body Weight and Caloric Efficiency

**Figure 2:**
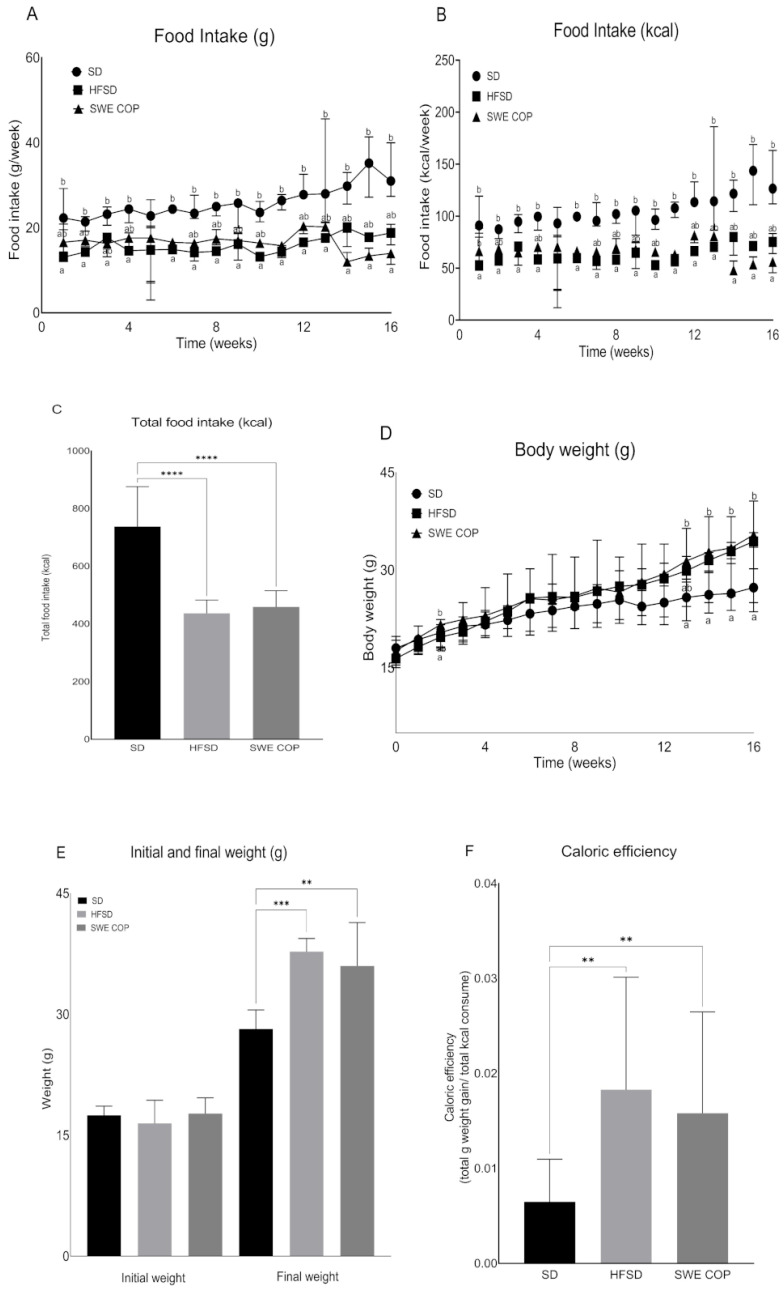
Effects of the diet and SWE COP supplementation on food and energy intake, weight gain and caloric efficiency. (**A**) Food intake was measured as g per cage per week by a subtractive method of weekly provision minus weekly remnants (**B**) and kcal consumed per week. (**C**) Total food intake (kcal). (**D**) Body weight was measured weekly (n = 6–7/diet). (**E**) Initial and final weights for each diet. (**F**) Caloric efficiency for each diet was calculated as total mg weight gain/total kcal consumed for the 16-week period. Values are represented as median and IR (n = 7/time point). Values with a different superscript letter (a, b) were significantly different by Kruskal–Wallis post hoc Dunn’s test (*p* ≤ 0.05). Data were compared by a one-way ANOVA followed by a Tukey’s multiple comparison test. **** *p* =< 0.0001; *** *p* = 0.0002; ** *p* = 0.003. Diets: SD = standard diet, HFSD = high-fat and high-sugar diet; SWE COP = subcritical water extract of California olive pomace.

During the first 13 weeks of the 16-week study, the SWE COP group had a food intake comparable to that of both the HFSD and SD groups; however, the control group with the standard diet (SD) consumed significantly more food than the HFSD group, except during weeks 5, 14, 15, and 16. In those final three weeks, a change in feeding behavior was observed, as the SWE COP–supplemented group exhibited a decrease in food intake, which nevertheless remained similar to that of the HFSD group (Figure 2A,B). At the end of the study, total caloric intake was significantly higher in the SD group compared with both the HFSD (737.2 ± 138.7 vs. 436.4 ± 45.93 kcal; *p* < 0.0001) and SWE COP (737.2 ± 138.7 vs. 458.6 ± 56.35 kcal; *p* < 0.0001) groups (Figure 2C). Despite the higher energy intake in the SD group, body weight gain was lower than in the HFSD and SWE COP groups. Initial body weights were 17.49 ± 1.11, 16.49 ± 2.86, and 17.69 ± 1.95 g for the SD, HFSD, and SWE COP groups, respectively. After sixteen weeks, final body weight was significantly higher in the HFSD (37.77 ± 1.63 g; *p* = 0.001) and SWE COP (36.00 ± 5.38 g; *p* = 0.004) groups than in the SD group (28.18 ± 2.37 g) (Figure 2D,E). Caloric efficiency, defined as total grams of weight gained per total kilocalories consumed, was significantly greater in both the HFSD and SWE COP groups (Figure 2F).

#### 2.3.2. Effects of SWE COP on Glucose Tolerance

**Figure 3:**
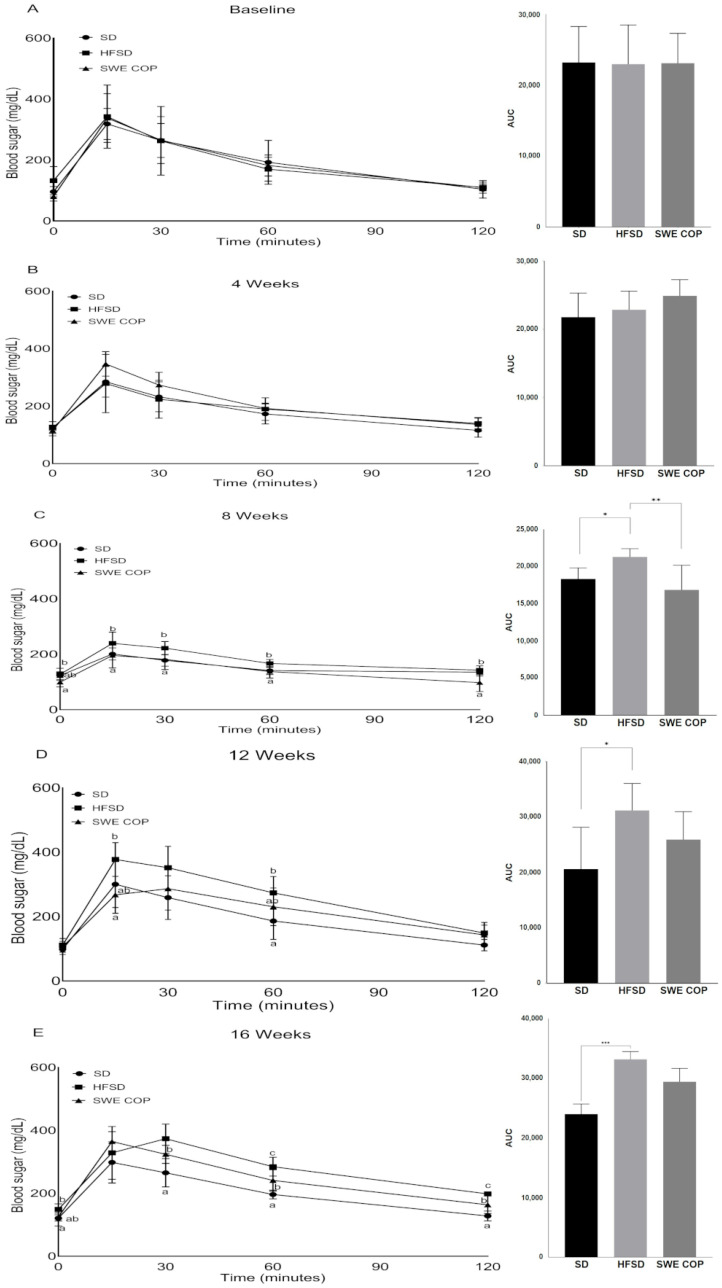
Effects of the diet and SWE COP supplementation on glucose tolerance. Glucose tolerance tests were performed prior to the feeding regimen (**A**) and after Weeks 4 (**B**), 8 (**C**), 12 (**D**), and 16 (**E**) of the diet. Values are represented as mean ± SEM (n = 7/time point). Data were compared using one-way ANOVA and Tukey’s test. Values with a different superscript letter (a, b, c) were significantly different. *** *p* = 0.0002; ** *p* = 0.003; * *p* < 0.05. Diets: SD = standard diet, HFSD = high-fat and high-sugar diet; SWE COP = subcritical water extract of California olive pomace.

To evaluate the impact of SWE COP supplementation on glucose metabolism under the HFSD diet, an intraperitoneal glucose tolerance test (IGTT) was conducted at key intervals. Before the intervention and at Week 4, no significant differences were observed in fasting glucose levels among the groups (Figure 3A,B). At Week 4, the SWE COP supplementation effectively prevented the early glucose excursions observed in the HFSD group. Glucose levels in the SWE COP group (e.g., 195.5 ± 16.9 mg/dL at 15 min) were comparable to those observed in the SD control group (200.9 ± 8.1 mg/dL) and significantly lower than those of the HFSD group (239.3 ± 16.6 mg/dL; *p ≤* 0.05). Correspondingly, the area under the curve (AUC) for the SWE COP group was significantly lower than that of the HFSD group (16,875 ± 1259 vs. 21,321 ± 455; *p* = 0.004), indicating an early stabilization of glucose tolerance (Figure 3C). By Week 12, the metabolic challenge of the HFSD became more evident. While the SD group maintained the lowest overall AUC (20,599), it exhibited a sharp initial glucose peak at 15 min (377.3 mg/dL), which was transiently higher than the HFSD and SWE COP groups. This is attributed to the faster glucose absorption and subsequent robust insulin response typical of lean models (Figure 3D). At the final checkpoint, Week 16, the HFSD group showed a marked deterioration in glucose clearance. Although total AUC differences between SWE COP and HFSD did not reach statistical significance at this stage, a clear mitigating effect was observed at the 60 min mark: SWE COP glucose levels were significantly lower than the HFSD group (240.8 vs. 284.5 mg/dL; *p* = 0.03), although still higher than the SD control (196.1 mg/dL; *p* = 0.02). This suggests that while SWE COP does not fully normalize glucose kinetics to SD levels after prolonged high-fat feeding, it successfully moderates the severity of hyperglycemia (Figure 3E).

#### 2.3.3. Effects of SWE COP on Insulin Tolerance

**Figure 4:**
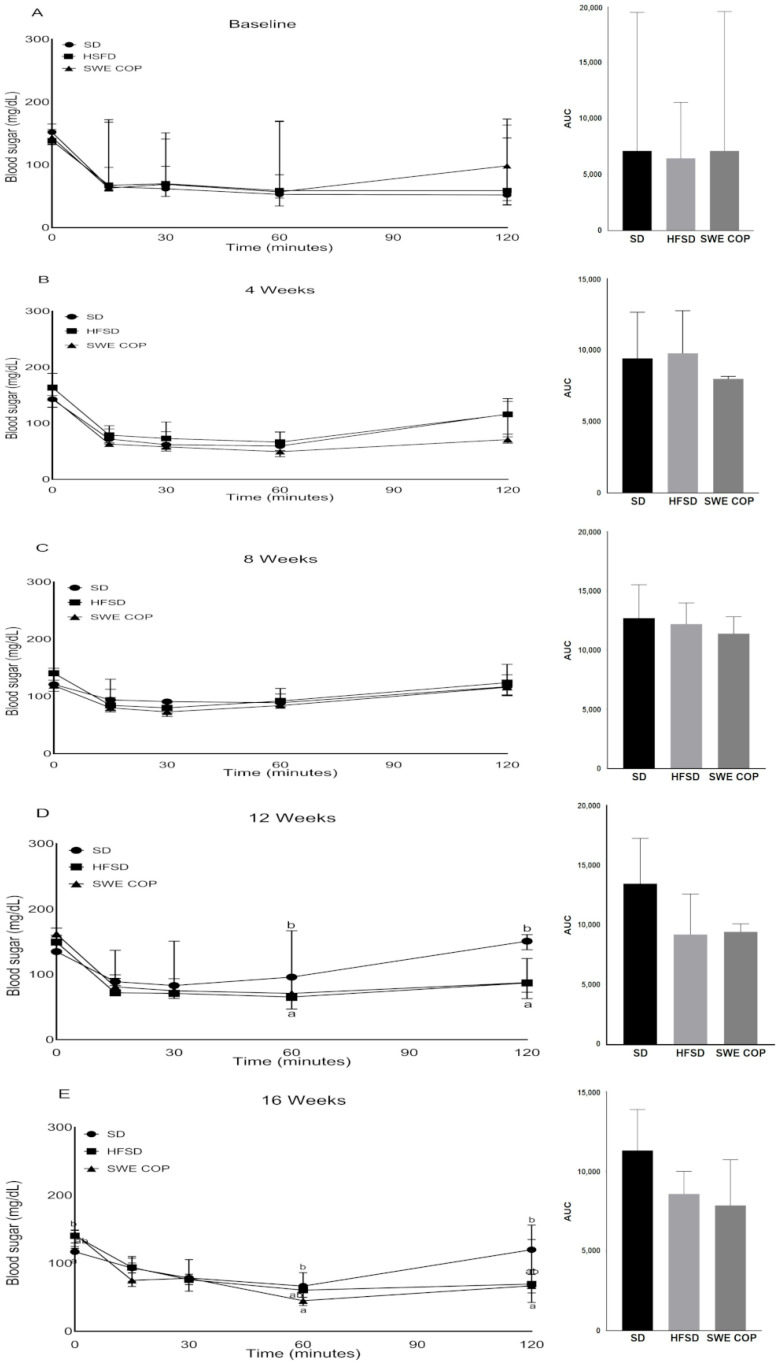
Effects of the diet and SWE COP supplementation on insulin tolerance. Insulin tolerance tests were performed prior to the feeding regimen (**A**) and after Weeks 4 (**B**), 8 (**C**), 12 (**D**), and 16 (**E**) of the diet. Values are represented as median ± SEM (n = 7/time point). Data were compared by Kruskal–Wallis followed by Dunn’s test. Values with a different superscript letter (a, b) were significantly different. Diets: SD = standard diet, HFSD = high-fat and high-sugar diet; SWE COP = subcritical water extract of California olive pomace.

During the insulin tolerance tests, at Week 4, no significant differences in insulin response were observed between the groups, indicating that insulin sensitivity remained intact during the early phase of the dietary intervention (Figure 4A–C). However, by Week 12, the HFSD group began to show signs of incipient insulin resistance, evidenced by a slower rate of glucose clearance compared to the SD group. At this stage, SWE COP supplementation showed a protective trend, maintaining glucose levels closer to the SD group than to the HFSD group. By Week 16, the metabolic divergence was fully established. The HFSD group exhibited a marked impairment in insulin-stimulated glucose disposal. Although the SD group displayed the highest insulin sensitivity, it also showed transiently higher glucose values at later checkpoints (e.g., 90–120 min) compared to earlier points in the same test (71 vs. 96 mg/dL, *p* = 0.02 and 87 vs. 151 mg/dL, *p* = 0.01) (Figure 4D,E).

#### 2.3.4. Effects of SWE COP on Serum Triglyceride (TG), Total Cholesterol (TC), and c-HDL

**Figure 5:**
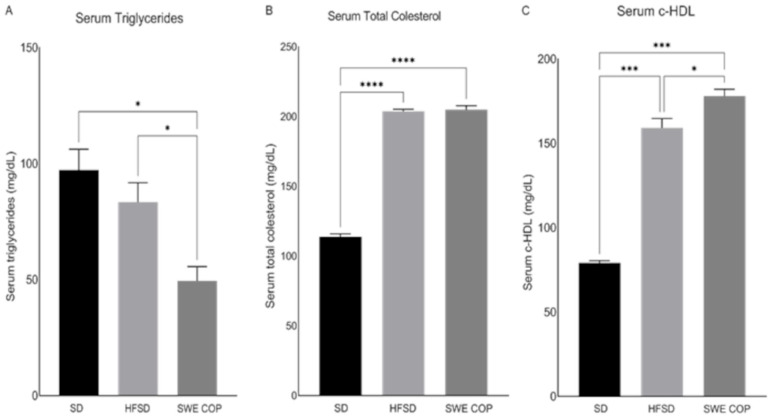
Effects of the diet and SWE COP supplementation on lipid profile. Serum triglycerides (**A**), total cholesterol (**B**) and c-HDL (**C**). Values are represented as mean ± SEM. Data were compared by one-way ANOVA followed by Tukey’s test. * (*p* ≤ 0.04); *** (*p* ≤ 0.0006); **** (*p* ≤ 0.0001). Diets: standard diet; SD (n = 7); high-fat and high-sugar diet; HFSD (n = 7); subcritical water extract of California olive pomace; SWE COP (n = 7).

After 16 weeks, the SWE COP-supplemented group showed lower TG levels compared with both the HFSD and SD groups (49.50 ± 4.30, 83.40 ± 5.90 and 97.25 ± 6.35 mg/dL, respectively) (*p* = 0.04) (Figure 5A). The TC concentrations were 205.05 ± 1.95, 203.8 ± 1.00 and 113.0 ± 1.50 in SWE COP, HFSD and SD groups, respectively. Statistically significant differences were observed between the SWE COP and SD groups and between the HFSD and SD groups (*p* =< 0.0001) (Figure 5B). c-HDL levels were 178.05, 159.2 and 79.15 in SWE COP, HFSD and SD groups, respectively. Statistically significant differences were observed, with significant increases in both the SWE COP and HFSD groups compared with the SD group (*p* = 0.0004) (Figure 5C).

#### 2.3.5. Effects of SWE COP on the Heart, Kidney, Liver Mass and Adipose Tissue

**Figure 6:**
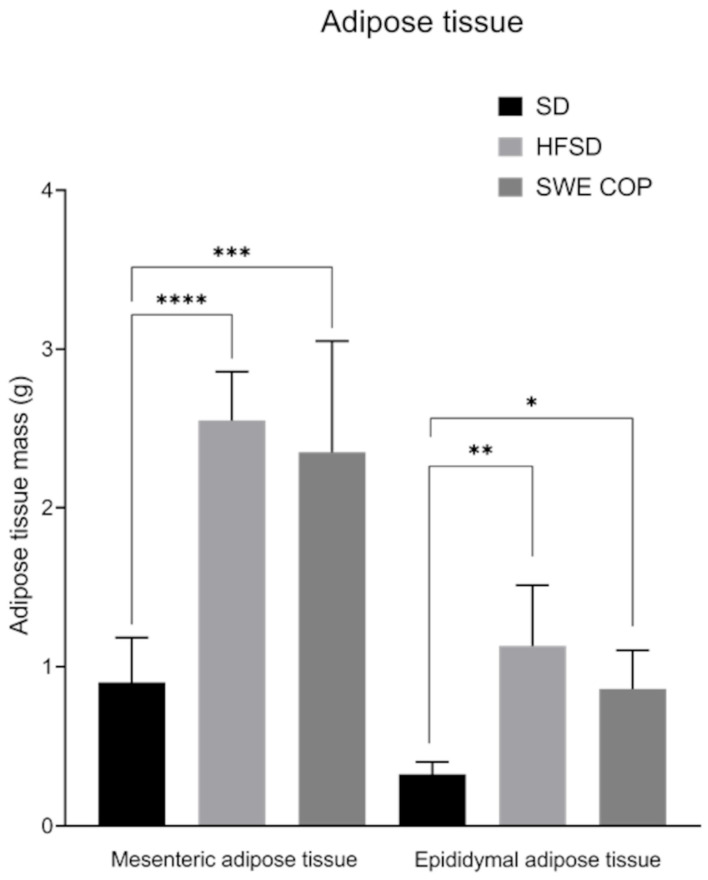
Effects of the diet and SWE COP supplementation on mesenteric and epididymal adipose tissue. Mesenteric adipose tissue. Data were compared by one-way ANOVA followed by Tukey’s multiple comparison test. **** (*p* =< 0.0001); *** (*p* = 0.0002); epididymal adipose tissue: ** (*p* = 0.003), * (*p* = 0.01). Diets: standard diet; SD (n = 7); high-fat and high-sugar diet; HFSD (n = 7); subcritical water extract of California olive pomace; SWE COP (n = 7).

No statistically significant differences were observed in heart, kidney, or liver weights among the experimental groups. However, mesenteric and epididymal adipose tissue weights were lower in the SD group compared with both the SWE COP and HFSD groups (*p* =< 0.0001) (Table 3) (Figure 6).

#### 2.3.6. Effects of SWE COP on Liver Parenchymal Morphology

**Figure 7:**
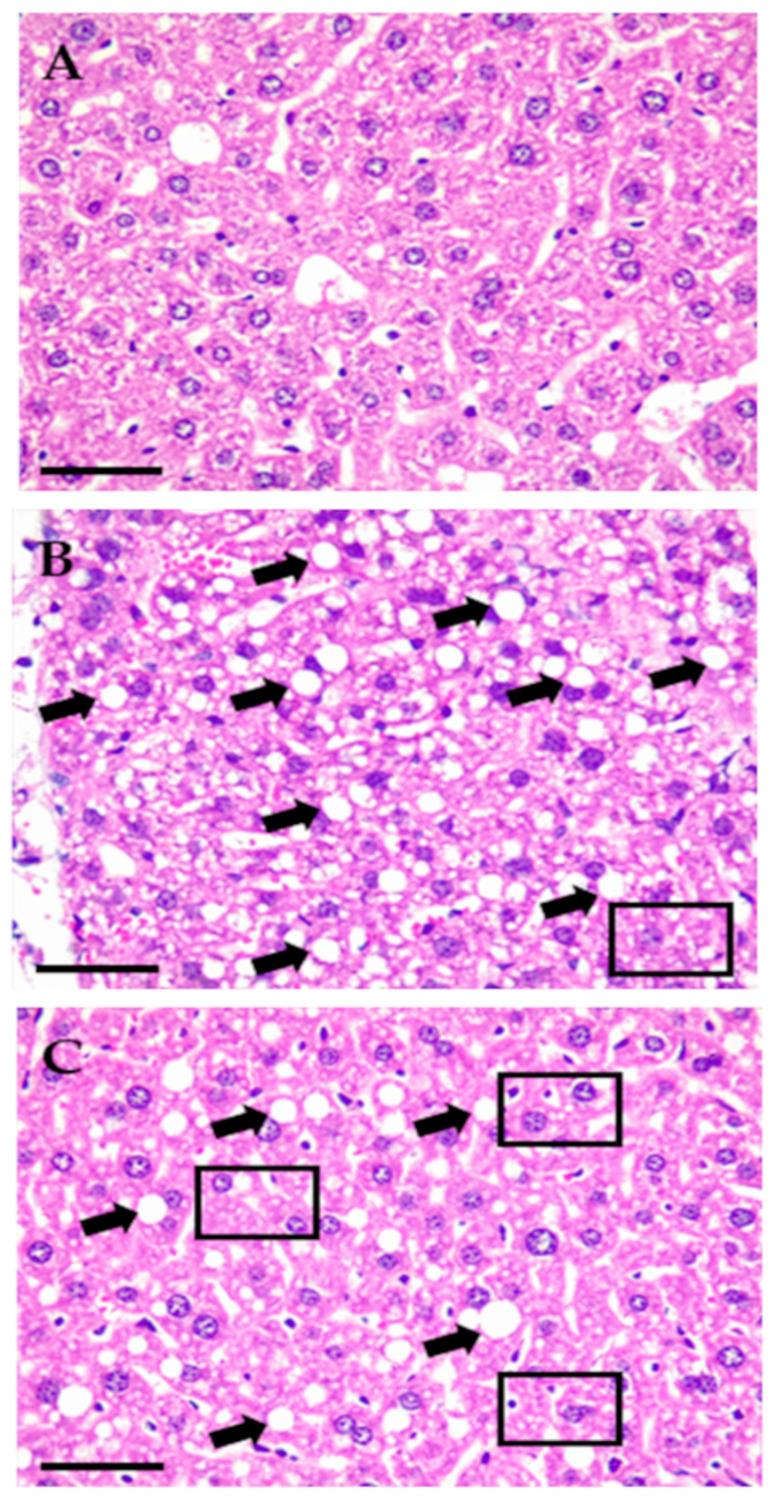
Effects of the diet and SWE COP on liver parenchymal morphology. (**A**) = SD, (**B**) = HFSD, (**C**) = SWE COP. Diets: standard diet; SD (n = 7); high-fat and high-sugar diet; HFSD (n = 7); subcritical water extract of California olive pomace; SWE COP (n = 7). Rectangles = cells with microvesicular steatosis, black arrows = macrovesicular steatosis. Scale bar = 50 µm.

In the SD group (Figure 7A), liver parenchyma exhibited a normal architecture characterized by polyhedral hepatocytes in linear cords, with a low presence of lipid deposits corresponding to a NASH CRN score of zero [22] and without the presence of an inflammatory response or of apoptotic or necrotic hepatocytes. In contrast, the HFSD group (Figure 7B) showed predominantly macrovesicular steatosis, both within and around hepatocytes, diffusely distributed throughout the liver parenchyma and, to a lesser extent, microvesicular steatosis, corresponding to a NASH CRN score of 3. The SWE COP-supplemented group (Figure 7C) displayed a reduced degree of macrovesicular steatosis compared with the HFSD group but a higher proportion of microvesicular steatosis, resulting in an intermediate NASH CRN score of 2.

## 3. Discussion

Olive pomace (OP) is the most important byproduct of olive oil production, and it contains a wide range of phenolic compounds with potential applications as a nutritional and functional food ingredient [11]. The antioxidant capacity of polyphenols is closely related to their chemical structure, as these compounds prevent the formation of free radicals involved in autoxidation processes by donating hydrogen electrons or atoms [23,24]. In this study, SWE was employed as an environmentally friendly extraction method that utilizes water instead of organic solvents, thereby improving the safety and sustainability of the resulting extracts for human consumption [25]. The chemical composition of subcritical water extract of olive pomace (SWE COP) revealed that carbohydrates, including soluble and insoluble fibers, were the major components. The SWE COP showed a TSP content of 6%, which was higher than that reported for extracts obtained by pressurized liquid extraction [26] and ultrasound-assisted extraction from Spanish olive pomace [27]. At higher temperatures, the penetration of the subcritical water is generally easier, and the dissolution of the compounds is faster, resulting in higher TPC values [28]. In addition, when the water in a subcritical state is used as a solvent, it might recover polar and non-polar compounds. SWE uses pressure in a range of 10–80 bar, which allows the penetration of the solvent through the pores of the matrix, explaining the greater concentration of phenolic compounds in our extract [29,30]. The use of plant-derived polyphenols is considered promising for the prevention of metabolic disorders such as obesity [31], type 2 diabetes [32], and dyslipidemia [33]. The individual phenolic analysis of SWE COP showed that the most abundant phenolic compounds were 3,4-DHPEA-EDA, hydroxytyrosol and verbascoside. Several studies have demonstrated the benefits of hydroxytyrosol supplementation in metabolic syndrome parameters [34,35,36,37]. More recently, verbascoside has been shown to be effective in treating atherosclerotic complications and dyslipidemia [38], as well as certain neurological disorders [39].

In the present study, both the SWE COP and HFSD groups (receiving ~50% of calories from fat) exhibited lower food and caloric intake compared with the SD group. This observation may be attributed, according to the literature, to appetite regulation via the gut–brain axis (GBA). The crosstalk between the gastrointestinal tract and the central nervous system is mediated by key anorexigenic hormones, such as glucagon-like peptide 1 (GLP-1), cholecystokinin (CCK), and peptide YY (PYY), which are secreted in response to specific nutrients [40]. High-fat diets, in particular, are known to promote satiety by stimulating the release of these hormones [41]. Our findings align with previous reports, such as those involving mulberry leaf polyphenols, where supplementation did not significantly alter food intake compared to unsupplemented high-fat controls [42].

Regarding body composition, while both high-fat groups showed greater weight gain than the SD group, no statistically significant differences in total body weight were observed between the SWE COP-supplemented mice and the HFSD group. Consequently, the effects of the extract should be interpreted as metabolic modulation rather than anti-obesity per se. Nevertheless, the SWE COP group displayed a non-significant trend toward reduced weight gain and, notably, a lower caloric efficiency compared to the HFSD group. This suggests that despite similar caloric intake and total body mass, the supplemented mice utilized energy differently. This phenomenon is often associated with increased energy expenditure, potentially linked to molecular pathways such as the up-regulation of UCP1 and the activation of AMPK, which drive fatty acid oxidation and the browning of the white adipose tissue [43]. Although gene expression was not measured in this study, the shift in caloric efficiency suggests that the SWE COP may favor a more favorable metabolic phenotype independent of substantial changes in total body weight.

Crucially, SWE COP supplementation significantly moderated the insulin resistance induced by the high-fat diet. While the supplemented group did not return to the baseline levels of the SD group, it achieved a statistically significant improvement in glucose-disposal rates compared to the HFSD group. These results, integrated over the 16-week period, demonstrate that SWE COP acts as a metabolic mitigator, partially preserving insulin signaling integrity despite the continuous obesogenic challenge. Several mechanisms have been proposed in the literature to explain the beneficial effect of polyphenols on insulin sensitivity. These include enhanced responsiveness of peripheral tissues to insulin-stimulated glucose uptake, mediated by GLUT4 translocation to the plasma membrane, as well as modulation of incretin hormones, such as glucagon-like peptide-1 (GLP-1). This hormone promotes insulin release induced by postprandial glucose levels. Through the activation of signal-regulated kinase (ERK)1/2, Ca^2^+ influx, reduction of nuclear Foxo1 localization, and reduction of AKT phosphorylation, polyphenols have also been implicated in interfering with incretin hormones, such as glucagon-like peptide-1 (GLP-1). This hormone promotes insulin release induced by postprandial glucose levels when it is released by L cells in the distal small intestine and colon [44]. Delgadillo-Puga et al. observed, as in our study, that the pecans’ polyphenols enhanced glucose tolerance by 37%, likely by preventing pancreatic islet hypertrophy and improving glucose metabolism and insulin sensitivity [45].

Regarding lipid metabolism, the SWE COP reduced plasma triglyceride levels while increasing c-HDL, consistent with previous studies using pecans’ polyphenols. These effects may be related to the up-regulation of UCP-1 in brown adipose tissue, which promotes lipid oxidation and indirectly attenuates fat accumulation and the up-regulation of p-AMPK and p-AKT in muscle tissue. The activation of p-AMPK stimulates mitochondrial biogenesis, thereby enhancing lipid oxidation and attenuating fat accumulation [45]. Our results are also consistent with a recent study on Western diet-fed mice supplemented with 1% and 3% polyphenol-rich extracts, where polyphenols inhibited intestinal lipid absorption [46].

The effect of SWE COP on organ and tissue weights was particularly evident in adipose tissue. Both mesenteric and epididymal adipose tissue weights were lower in the supplemented group compared with the non-supplemented HFSD group. As discussed above, this may be due to the ability of polyphenols to attenuate obesity-induced hepatic steatosis by enhancing lipolysis and inhibiting lipogenesis, as well as reducing triglyceride accumulation and suppressing the expression of adipogenic genes, such as adiponectin, nuclear receptor, PPARγ, and fatty acid binding protein (FABP4) in adipocytes. Moreover, dietary polyphenols increase the formation of beige adipocytes, thereby reducing adipocyte density or stimulated cellular energy expenditure and reducing the accumulation of iWAT through the induction of beige adipose tissue (BAT) differentiation [47].

These mechanisms likely contribute to the reduced severity of hepatic steatosis observed in the SWE COP–supplemented group, supporting the potential of subcritical water extracts from olive pomace as functional ingredients for mitigating metabolic alterations induced by obesogenic diets.

While the current findings demonstrate significant metabolic improvements, they also establish a strategic framework for future research. The observed phenotypic changes suggest strong nutrigenomic potential for the COP of SWE, as evaluated in studies similar to ours. In this regard, subsequent studies will focus on the direct quantification of key molecular markers, such as UCP1 for adipose tissue browning and GLUT4 for insulin signaling, to fully elucidate the underlying pathways. Furthermore, after establishing a solid baseline in male models to minimize hormonal confounding factors, future research with both sexes will be essential to validate the generalizability of these effects. Finally, the efficacy and safety profile observed with the 3% dose provides a clear rationale for future dose–response trials.

## 4. Conclusions

This study demonstrates that Subcritical Water Extraction (SWE) represents an effective and sustainable green technology for recovering phenolic-rich bioactive fractions from California olive pomace (COP). Phytochemical profiling identified 18 distinct phenolic compounds, whose combined and potentially synergistic effects contribute to a robust antioxidant capacity. Evidence from in vivo experimentation indicates that 16-week dietary supplementation with 3% (*w*/*w*) SWE COP attenuated the development of metabolic alterations induced by a high-fat and high-sugar diet (HFSD). Specifically, the extract improved glucose homeostasis and insulin sensitivity, while orchestrating a favorable shift in the lipid profile by reducing total cholesterol and triglycerides alongside a concomitant increase in HDL cholesterol levels.

These findings help bridge the gap between the chemical characterization of olive pomace polyphenols and their physiological relevance. Collectively, the results support the potential of SWE COP as a functional ingredient for incorporation into nutraceuticals and dietary strategies aimed at improving the bioavailability and bioaccessibility of olive-derived phenolic compounds, particularly in the context of metabolic dysfunctions associated with obesity.

## 5. Materials and Methods

### 5.1. Plant Material

Fresh Arbequina olive pomace (OP) from the first olive oil extraction was collected at California Olive Ranch in Artois, CA, during the 2023 harvest season and was stored at room temperature in sealed buckets for 4 h for transportation to the Western Regional Research Center in Albany, CA, USA. Then it was processed following the steps reported by Zhao et al. [48,49].

Fresh first oil extraction OP was steam-blanched for enzymatic inactivation to reduce phenolic losses. Blanching was conducted using a steam blancher at atmospheric pressure over 0.25” thick olive pomace to a final temperature of 80 °C after 3 min. The separation of skins and pits was conducted using a 150 Langsenkamp Laboratory Separator (Warner Bodies, Elwood, IN, USA). The pomace was passed through the separator in two stages. First, using a 0.060-inch hole diameter S.S. screen and then using a 0.027-inch hole diameter S.S. screen. The pitted olive pomace was drum-dried on a Buflovaks Atmospheric Double Drum Dryer (Hebeler Process Solutions, Tonawanda, NY, USA), at a space of 9–10/1000” at 135 °C. Drum-drying treatments were differentiated by rotational drum speeds of 92 s/rev. To obtain smaller particle sizes, drum-dried OP samples were milled for 6 s with a KRUPS F203 (KRUPS, Parsippany, NJ, USA) coffee mill.

### 5.2. Subcritical Water Extraction (SWE)

The extraction of US California pitted, drum-dried Arbequina OP was performed using an ASE 350 Accelerated Solvent Extractor (Dionex Corporation, Sunnyvale, CA, USA) equipped with a solvent controller unit. Extractions were processed within an experimental design of response surface regression (RSR) with three factors and three levels: temperature, time and running cycles [49,50,51]. The optimum extraction conditions were: a temperature of 140 °C, a static time of 10 min and 2 running cycles.

### 5.3. Chemicals

Analytical-grade 2,2-Diphenyl-1-picrylhydrazyl radical (DPPH) and Trolox^®^ were purchased from Fisher Scientific (Waltham, MA, USA). In addition, 96–98% (g/g) concentrated sulfuric acid, Folin–Ciocalteu reagents, sodium carbonate, phenolic compound standards of gallic acid, hydroxytyrosol, tyrosol, 4-hydroxyphenylacetic acid (4-HPA), vanillic acid, vanillin, o-coumaric acid, oleuropein, prinoresinol, cinnamic acid, caffeic acid, p-coumaric acid, ferulic acid, apigenin-7-glucoside, apigenin, luteolin-7-glucoside and luteolin were purchased from Sigma-Aldrich (St. Louis, MO, USA). Verbascoside was bought from HWI Group (Ruelzhelm, Germany). Rutin was bought from PhytoLab GmbH & Co. KG (Vestenbergsgreuth, Germany)

### 5.4. Basic Chemical Composition Analysis

#### 5.4.1. Crude Protein Content

The protein content of the SWE OP was measured using an FP-628 TrueSpec N analyzer (LECO Corporation, St. Joseph, MI, USA), which followed the method of Zhao et al. [48]. One gram of SWE OP was placed into a ceramic boat for the combustion process. Each sample was analyzed in triplicate, and the nitrogen content of the SWE OP was multiplied by a factor of 6.25 to convert it into protein content.

#### 5.4.2. Oil Content

In total, 10 g of SWE OP was extracted by hexane Soxhlet extraction [52] in a Universal Extractor (BÜCHI, New Castle, DE, USA). Each sample was processed in triplicate using 30 cycles of solvent extraction.

#### 5.4.3. Ash Content

In total, 3 g of each sample was placed in triplicate in crucibles and introduced into a Lindberg/Blue M box furnace (Thermo Fisher Scientific, Waltham, MA, USA) at 550 °C for 16 h, following Zhao et al. with some modifications [53]. Before the final weighting, the crucibles containing the ash were carefully relocated in a desiccator to reach room temperature.

#### 5.4.4. Total Carbohydrates

Total carbohydrates were estimated by difference.

#### 5.4.5. Fiber

Soluble and insoluble dietary fiber content was measured by Medallion Labs (Medallion Laboratories, Minneapolis, MN, USA) based on fiber method AOAC 991.43 with modifications [54].

### 5.5. Total Phenolic Content Analysis

The total phenol content was determined by the Folin–Ciocalteu assay, and 30 µL of the extracted sample was added to 1.8 mL DI water, 150 µL Folin–Ciocalteu Reagent, 450 µL 20% (g/mL) NaCo, and 570 µL DI water (the adding sequence cannot be changed). This total 2 mL solution was incubated for 30 min in the dark. Then, 200 µL solution out of the 3 mL was pitted into a FALCON tissue culture plate with 96 wells (Corning Incorporated, Corning, NY, USA) and determined by absorption at 725 nm in a BioTek^®^ Sinergy H1 microplate reader (Winooske, VT, USA). Approximately 0.1–2.0 mg/mL of gallic acid equivalents (mg GAE/g DM, mg gallic acid/g of dry defatted matter) was used, and 30 µL of extraction solvent with the other chemical reagents served as the blank [55].

### 5.6. Analysis of Individual Phenolic Compounds

The identification of individual phenolic compounds was implemented by high-performance liquid chromatography (HPLC) using a diode-array detector (DAD) and by HPLC-electrospray ionization (ESI)-quadrupole-time-of-flight mass spectrometry (Q-ToF-MSn) analysis using an Agilent 1290 high-performance liquid chromatography (HPLC) system (Santa Clara, CA, USA) with an Agilent 1290 diode-array detector (DAD), following Sinrod et al. [8]. An analytical C18 column (Eclipse Plus, 4.6 mm × 250 mm, 5 µm, Agilent Technologies) was used for separation. Elution was applied using mobile phase A (3% acetic acid aqueous solution) and mobile phase B (50% methanol and 50% acetonitrile). The following linear gradient was used: 5% B to start (with 95% A, similarly hereinafter); a linear increase to 30% B at 25 min, to 35% B at 35 min, to 40% B at 40 min, to 70% B at 50 min, to 100% B at 55 min; a decrease to 5% B at 60 min and a hold at 5% B for 5 min for the column equilibrium for the next injection. The flow rate was 1.0 mL/min. The injection volume was 20 µL. The DAD was set to absorbance wavelengths at 280 nm for hydroxytyrosol, tyrosol, 4-hydroxyphenylacetic acid (4-HPA), vanillic acid, vanillin, o-coumaric acid, oleuropein, pinoresinol, and cinnamic acid; at 320 nm for caffeic acid, p-coumaric acid, ferulic acid, apigenin-7-glucoside, apigenin, and verbascoside; and at 365 nm for rutin, luteolin-7-glucoside, and luteolin. Standard curves were made using each of the standard chemicals at concentrations of 10, 20, 40, 60, 80 and 100 mg/L, respectively.

### 5.7. Ethics Statement

All experiments were performed in accordance with the guidelines of the Mexican regulations (NOM-062-ZOO-1999), after approval of the experimental protocol by the local Ethics committee of the Center for Research and Teaching in Health Sciences of the Universidad Autónoma de Sinaloa. Every precaution was taken to minimize stress and the number of animals used in each series of experiments.

### 5.8. Animals and Study Design

Four-week-old male C57BL/6N mice (Círculo ADN, S.A de C.V. Ciudad de México) (n = 20) were maintained in a 12 h light/dark cycle at 28 ± 1 °C and 60 ± 5% humidity with free access to water and food ad libitum. Initially, 21 animals were randomized in three groups: mice fed a commercial normcaloric diet (Rodent Laboratory Diet 5001), approximately 5% calories from fat (SD, n = 7), mice fed HFSD, approximately 50% calories from fat (HFSD, n = 7), and mice fed HFD, with 3% (*w*/*w*) subcritical water extract of California olive pomace (SWE COP) added (SWE COP, n = 7). In this study, a single dose of SWE COP was selected in a preliminary palatability trial. The high-fat and high-sugar diet was prepared according to the specifications of Chehade et al. (Table 4) with some adaptations to locally sold ingredients but respecting the same macro and micronutrient profile [56]. At the end of the treatment, sixteen weeks later, the animals were anesthetized with phenobarbital and sacrificed by cardiac puncture after 12 h of overnight fasting. Body weight, food, and energy intake were recorded at weekly intervals. In particular, body weight was analyzed as a repeated measure.

#### 5.8.1. Body Weight, Food Consumption, and Calorie Intake Estimation

The body weights of the mice were recorded once a week with a precision electronic scale (BAPRE-3, Rhino, CDMX, Mexico). To measure the body weight, the mice were positioned individually inside a metal basket placed on a tared scale, and the results were expressed in grams (g). Food consumption was measured daily on a precision electronic scale (BAPRE-3). To calculate food consumption, the amount of food placed in the cage was recorded, and the leftover food was measured on the previously tared scale the next day. To determine the amount consumed, the initially given amount of food was subtracted from the amount of leftover food. The dietary calories of the SD group were measured with the information provided by the manufacturer of each macronutrient. For HFSD and HFSD SWE COP, the caloric content was estimated according to the Atwater system used to determine the total calorific value of food by employing the 4-9-4 method. This system applies energy conversion factors to the macronutrients carbohydrate, fat, protein and fiber. The average values of energy are expressed as the number of calories per 1 g of the macronutrient. The Atwater general factor system includes energy values of 4 kcal per gram (kcal/g) (17 kJ/g) for protein, 4 kcal/g for carbohydrates and 9 kcal/g (37 kJ/g) for fat.

#### 5.8.2. Insulin Resistance Assessment

Intraperitoneally glucose tolerance test (IGTT): the mice were fasted for 12 h and orally infused with glucose (2 g/kg). Blood was collected from a tail vein and its glucose level was measured with a glucometer (Accu-Chek Performa test strips, Roche, Basel, Switzerland) before (0 min) and after intraperitoneal injection of glucose solution (15, 30, 60 and 120 min) [57]. The area under the curve (AUC) was calculated by nonlinear regression.

Intraperitoneally insulin tolerance test (IITT): the mice were fasted for 12 h and received an intraperitoneal injection of insulin solution (0.75 U/kg) [58]. A blood glucose test and AUC value calculation were carried out as for IGTT.

#### 5.8.3. Mouse Blood and Organ Collection

After 12 weeks of feeding with the four different diets, the mice were prepared for euthanasia. Blood samples were collected by cardiac puncture in tubes. The serum was separated by centrifugation at 5000 rpm for 10 min and then stored at −80 °C in an ultralow temperature freezer (Forma 900 Series, Thermo Fisher, Waltham, MA, USA) until analysis.

#### 5.8.4. Mice Blood and Organ Analysis

Glucose was measured every four weeks from blood obtained from the mice’s tails using a glucose test strip (Accu-Chek Performa test strips, Roche, Basel, Switzerland) and a blood glucometer (Accu-Chek Performa II, Roche, Basel, Switzerland).

Visceral adipose tissue and organs such as the liver, heart, and kidneys were removed from the mice to later be weighed by a Sartorius TE64 Talent Analytical Balance (Sartorius, Gottingen, Germany) and a watch glass; the scale was tared with the watch glass. The organs were weighed, and the obtained results were expressed in grams.

Cholesterol (total cholesterol and HDL cholesterol), triglycerides and hepatic enzymes were analyzed with a commercial assay kit (ELITechGroup Puteaux, France), following the manufacturer’s instructions.

#### 5.8.5. Histopathological Examination

After sacrifice, the livers were dissected, fixed in formalin (formaldehyde 10% 100 mL/L (J.T. Baker), NaH_2_PO_4_ 4 g/L (Vetec), Na_2_HPO_4_ 6.5 g/L (Fermont), and distilled water 900 mL, pH 7.4), dehydrated, clarified, embedded in paraffin (Leica Paraplast), and cut into 5 μm tissue sections using a microtome (Leica RM 2145 RTS) [59]. After deparaffination and hydration, tissue sections were stained with H&E. The histological sections were independently evaluated by two specialized pathologists. To ensure an unbiased assessment, a double-blind protocol was implemented, in which the observers remained unaware of the experimental group assignments during the microscopic examination. The evaluations were carried out with light microscopy (ZEISS Primo Star LED, Carl Zeiss, Gottingen, Germany). The most representative images were taken using Zen Zeiss imaging blue edition software (Carl Zeiss, Gottingen, Germany). The non-alcoholic steatohepatitis (NASH) degree was determined according to the histological score system of the clinical research network of NASH [22].

### 5.9. Statistics

Data were analyzed in triplicate by multiple comparison tests with Fisher’s least significant difference (LSD, *p* < 0.05) method by R software 4.1.2. Data normality and homogeneity of variance were assessed using the Shapiro–Wilk and Brown–Forsythe tests, respectively. For datasets following a normal distribution, statistical significance was analyzed by one-way ANOVA followed by Tukey’s multiple comparison test, and data are presented as mean ± SEM. For non-normally distributed data, the Kruskal–Wallis H test followed by Dunn’s post hoc test was used, and results are expressed as median and interquartile range (IQR). All analyses were performed using GraphPad Prism version 9.0 (GraphPad Software version 9.0, San Diego, CA, USA). A *p*-value < 0.05 was considered statistically significant.

## Figures and Tables

**Table 1:** Basic chemical analysis of SWE COP.

SWE COP Nutritional Component	% on Dried Basis
Protein	6.93
Fat	3.38
Minerals (ash)	16.88
Total Carbohydrates	72.81
Soluble Fiber	1.94
Insoluble Fiber	14.78
Other Carbohydrates	56.08
SWE COP: Subcritical Water Extract California Olive Pomace	

**Table 2:** Phenolic compounds in SWE COP.

TSP (mg AGE/g Freeze-Dried Powder)	66.62 ± 1.22
Individual Phenolics (mg/g) Freeze Dried SWE COP	
3,4-DHPEA-EDA in Ole equation	36.66 ± 0.03
Hydroxytyrosol	6.54 ± 0.00
Verbascoside	2.97 ± 0.00
4-HPA	2.31 ± 0.01
Hydroxytyrosol-glucoside	1.55 ± 0.00
Rutin	1.50 ± 1.55 × 10^−5^
Tyrosol-glucoside	1.32 ± 0.00
Tyrosol	0.80 ± 0.01
Oleuropein	0.64 ± 0.90
Unknown-1 in HT equation	0.34 ± 0.00
Luteolin-7-glucoside	0.20 ± 0.00
Vanillin	0.16 ± 0.00
p-coumaric acid	0.15 ± 0.00
Gallic acid	0.11 ± 0.02
Apigenin-7-glucoside	0.10 ± 0.03
Luteolin	0.09 ± 0.00
Caffeic acid	0.05 ± 4.8 × 10^−5^
Cinnamic acid	0.03 ± 0.00

**Table 3:** Heart, kidney, liver, mesenteric and epididymal adipose tissue.

Tissues	SD	HFSD	SWE COP
Heart (g)	0.138 ± 0.02	0.168 ± 0.02	0.164 ± 0.01
Kidney (g)	0.385 ± 0.05	0.411 ± 0.05	0.444 ± 0.05
Liver (g)	1.278 ± 0.18	1.421 ± 0.21	1.309 ± 0.18

Values are represented as mean ± SEM. Data were compared by one-way ANOVA followed by Tukey’s test. Diets: standard diet; SD (n = 7); high-fat and high-sugar diet; HFSD (n = 7); subcritical water extract of California olive pomace; SWE COP (n = 7).

**Table 4:** Composition of the high-fat diet (g/kg diet) based on the AIN-93 recommendations for protein, inorganic nutrients and fiber.

HFSD		HFSD + SWE COP	
Ingredient	g/kg	Ingredient	g/kg
Casein	83	Casein	83
L-cysteine	1.8	L-cysteine	1.8
Whole wheat flour	374.77	Whole wheat flour	374.77
High fructose corn syrup	264	High fructose corn syrup	264
Maltodextrin	60	Maltodextrin	60
Coconut oil	59.5	Coconut oil	59.5
Soybean oil	17.3	Soybean oil	17.3
Corn oil	10.6	Corn oil	10.6
Sunflower seeds oil	20.9	Sunflower seeds oil	20.9
Lard	18	Lard	18
Beef tallow	16	Beef tallow	16
Unsalted butter	23.3	Unsalted butter	23.3
Salt	4.0	Salt	4.0
Mineral mix	35	Mineral mix	35
Vitamin mix	10	Vitamin mix	10
Choline bitartrate	1.4	Choline bitartrate	1.4
		SWE COP	30
**Macronutrient**	**% Kcal**	**Macronutrient**	**% Kcal**
Protein	12.2	Protein	12.73
Fat	49.2	Fat	49.22
Carbohydrate	38.6	Carbohydrate	38.82
Kcal/g	4.0	**Kcal/g**	4.2

HFSD: high-fat and high-sugar diet; SWECOP: subcritical water extract California olive pomace.

## Data Availability

The raw data supporting the conclusions of this article will be made available by the authors on request.

## Abbreviations

The following abbreviations are used in this manuscript:

3,4-DHPEA-EDA	Oleuropein-aglycone di-aldehyde
4-HPA	4-Hydroxyphenil acetate
AMPK	AMP Activated Protein Kinase
AUC	Area under the curve
BAT	Beige adipose tissue
CCK	Cholecystokinin
C-HDL	High-density lipoprotein cholesterol
FABP4	Fatty acid binding protein
GBA	Gut–brain axis
GLP1	Glucagon like peptide
HFSD	High-fat and high-sugar diet
HPLC-DAD	High-performance liquid chromatography—diode array detector
IpGTT	Intraperitoneally glucose tolerance test
IpITT	Intraperitoneally insulin tolerance test
MetS	Metabolic syndrome
NASH CRN	NASH Clinical Research Network
OP	Olive pomace
PYY	Peptide YY
SD	standard diet
SWE	Subcritical Water Extraction
SWE COP	Subcritical Water Extraction California Olive Pomace
TC	Total cholesterol
TG	Tryglycerides
WAT	White adipose tissue

## References

[1] Congdon P., Amugsi D. (2022). Editorial: The Obesity Epidemic: Causes, Context, Prevention. Front. Public Health.

[2] Ballena-Caicedo J., Zuzunaga-Montoya F.E., Loayza-Castro J.A., Bustamante-Rodríguez J.C., Vásquez Romero L.E.M., Tapia-Limonchi R., De Carrillo C.I.G., Vera-Ponce V.J. (2025). Global Prevalence of Insulin Resistance in the Adult Population: A Systematic Review and Meta-Analysis. Front. Endocrinol..

[3] Teng M.L., Ng C.H., Huang D.Q., Chan K.E., Tan D.J., Lim W.H., Yang J.D., Tan E., Muthiah M.D. (2023). Global Incidence and Prevalence of Nonalcoholic Fatty Liver Disease. Clin. Mol. Hepatol..

[4] Gutiérrez-Solis A.L., Datta Banik S., Méndez-González R.M. (2018). Prevalence of Metabolic Syndrome in Mexico: A Systematic Review and Meta-Analysis. Metab. Syndr. Relat. Disord..

[5] Noubiap J.J., Nansseu J.R., Lontchi-Yimagou E., Nkeck J.R., Nyaga U.F., Ngouo A.T., Tounouga D.N., Tianyi F.-L., Foka A.J., Ndoadoumgue A.L. (2022). Geographic Distribution of Metabolic Syndrome and Its Components in the General Adult Population: A Meta-Analysis of Global Data from 28 million Individuals. Diabetes Res. Clin. Pract..

[6] Alamnia T.T., Sargent G.M., Kelly M. (2023). Dietary Patterns and Associations with Metabolic Risk Factors for Non-Communicable Disease. Sci. Rep..

[7] Ren Y., Zhao H., Yin C., Lan X., Wu L., Du X., Griffiths H.R., Gao D. (2022). Adipokines, Hepatokines and Myokines: Focus on Their Role and Molecular Mechanisms in Adipose Tissue Inflammation. Front. Endocrinol..

[8] Masenga S.K., Kabwe L.S., Chakulya M., Kirabo A. (2023). Mechanisms of Oxidative Stress in Metabolic Syndrome. Int. J. Mol. Sci..

[9] Ojangba T., Boamah S., Miao Y., Guo X., Fen Y., Agboyibor C., Yuan J., Dong W. (2023). Comprehensive Effects of Lifestyle Reform, Adherence, and Related Factors on Hypertension Control: A Review. J. Clin. Hypertens..

[10] De Bacquer D., Astin F., Kotseva K., Pogosova N., De Smedt D., De Backer G., Rydén L., Wood D., Jennings C., for the EUROASPIRE IV and V surveys of the European Observational Research Programme of the European Society of Cardiology (2022). Poor Adherence to Lifestyle Recommendations in Patients with Coronary Heart Disease: Results from the EUROASPIRE Surveys. Eur. J. Prev. Cardiol..

[11] Difonzo G., Troilo M., Squeo G., Pasqualone A., Caponio F. (2020). Functional Compounds from Olive Pomace to Obtain High-Added Value Foods—A Review. J. Sci. Food Agric..

[12] Inzunza-Soto M., Thai S., Sinrod A., Olson D., Avena-Bustillos R., Li X., Rolston M., Wang S., Teran-Cabanillas E., Yokoyama W. (2021). Health Benefits of First and Second Extraction Drum-Dried Pitted Olive Pomace. J. Food Sci..

[13] Antónia Nunes M., Costa A.S.G., Bessada S., Santos J., Puga H., Alves R.C., Freitas V., Oliveira M.B.P.P. (2018). Olive Pomace as a Valuable Source of Bioactive Compounds: A Study Regarding Its Lipid- and Water-Soluble Components. Sci. Total Environ..

[14] Mansour H.M.M., Zeitoun A.A., Abd-Rabou H.S., El Enshasy H.A., Dailin D.J., Zeitoun M.A.A., El-Sohaimy S.A. (2023). Antioxidant and Anti-Diabetic Properties of Olive (Olea Europaea) Leaf Extracts: In Vitro and In Vivo Evaluation. Antioxidants.

[15] Xia H.-M., Wang J., Xie X.-J., Xu L.-J., Tang S.-Q. (2019). Green Tea Polyphenols Attenuate Hepatic Steatosis, and Reduce Insulin Resistance and Inflammation in High-Fat Diet-Induced Rats. Int. J. Mol. Med..

[16] Nwakiban Atchan A.P., Shivashankara S.T., Piazza S., Tchamgoue A.D., Beretta G., Dell’Agli M., Magni P., Agbor G.A., Kuiaté J.-R., Manjappara U.V. (2022). Polyphenol-Rich Extracts of Xylopia and Aframomum Species Show Metabolic Benefits by Lowering Hepatic Lipid Accumulation in Diet-Induced Obese Mice. ACS Omega.

[17] Huang F., Wang J., Yu F., Tang Y., Ding G., Yang Z., Sun Y. (2018). Protective Effect of Meretrix Meretrix Oligopeptides on High-Fat-Diet-Induced Non-Alcoholic Fatty Liver Disease in Mice. Mar. Drugs.

[18] Usman I., Hussain M., Imran A., Afzaal M., Saeed F., Javed M., Afzal A., Ashfaq I., Al Jbawi E., Saewan S.A. (2022). Traditional and Innovative Approaches for the Extraction of Bioactive Compounds. Int. J. Food Prop..

[19] Somat H.A., Thani N.M., Mustapha W.A.W., Lim S.J., Seng N.S.S., Rahman H.A., Razali N.S.M., Ali M.M., Kamal S.M.M. (2026). Subcritical Water Extraction of Bioactive Compounds from Plant Materials: Recent Advances. J. Future Foods.

[20] Cheng Y., Xue F., Yu S., Du S., Yang Y. (2021). Subcritical Water Extraction of Natural Products. Molecules.

[21] Rodríguez-Llorente D., Martín-Gutiérrez D., Suárez-Rodríguez P., Navarro P., Álvarez-Torrellas S., García J., Larriba M. (2023). Sustainable Recovery of Phenolic Antioxidants from Real Olive Vegetation Water with Natural Hydrophobic Eutectic Solvents and Terpenoids. Environ. Res..

[22] LaBrecque D.R., Abbas Z., Anania F., Ferenci P., Khan A.G., Goh K.-L., Hamid S.S., Isakov V., Lizarzabal M., Peñaranda M.M. (2014). World Gastroenterology Organisation Global Guidelines: Nonalcoholic Fatty Liver Disease and Nonalcoholic Steatohepatitis. J. Clin. Gastroenterol..

[23] Sakurai S., Kawakami Y., Kuroki M., Gotoh H. (2022). Structure–Antioxidant Activity (Oxygen Radical Absorbance Capacity) Relationships of Phenolic Compounds. Struct. Chem..

[24] Parcheta M., Świsłocka R., Orzechowska S., Akimowicz M., Choińska R., Lewandowski W. (2021). Recent Developments in Effective Antioxidants: The Structure and Antioxidant Properties. Materials.

[25] Koina I.M., Sarigiannis Y., Hapeshi E. (2023). Green Extraction Techniques for the Determination of Active Ingredients in Tea: Current State, Challenges, and Future Perspectives. Separations.

[26] Cea Pavez I., Lozano-Sánchez J., Borrás-Linares I., Nuñez H., Robert P., Segura-Carretero A. (2019). Obtaining an Extract Rich in Phenolic Compounds from Olive Pomace by Pressurized Liquid Extraction. Molecules.

[27] Contreras M.d.M., Gómez-Cruz I., Romero I., Castro E. (2021). Olive Pomace-Derived Biomasses Fractionation through a Two-Step Extraction Based on the Use of Ultrasounds: Chemical Characteristics. Foods.

[28] Žagar T., Frlan R., Kočevar Glavač N. (2024). Using Subcritical Water to Obtain Polyphenol-Rich Extracts with Antimicrobial Properties. Antibiotics.

[29] Ferreira C., Moreira M.M., Delerue-Matos C., Sarraguça M. (2023). Subcritical Water Extraction to Valorize Grape Biomass—A Step Closer to Circular Economy. Molecules.

[30] Shi F., Jiang Z.-B., Xu J., Bai X.-P., Liang Q.-Y., Fu Z.-H. (2022). Optimized Extraction of Phenolic Antioxidants from Red Pitaya (Hylocereus Polyrhizus) Seeds by Subcritical Water Extraction Using Response Surface Methodology. Food Meas..

[31] van der Zande H.J.P., Lambooij J.M., Chavanelle V., Zawistowska-Deniziak A., Otero Y., Otto F., Lantier L., McGuinness O.P., Le Joubioux F., Giera M. (2021). Effects of a Novel Polyphenol-Rich Plant Extract on Body Composition, Inflammation, Insulin Sensitivity, and Glucose Homeostasis in Obese Mice. Int. J. Obes..

[32] Sarkar D., Christopher A., Shetty K. (2022). Phenolic Bioactives from Plant-Based Foods for Glycemic Control. Front. Endocrinol..

[33] Alotaibi B.S., Ijaz M., Buabeid M., Kharaba Z.J., Yaseen H.S., Murtaza G. (2021). Therapeutic Effects and Safe Uses of Plant-Derived Polyphenolic Compounds in Cardiovascular Diseases: A Review. Drug Des. Dev. Ther..

[34] Harun N. (2025). Administration of Oleuropein or Hydroxytyrosol Improves Diabetic Markers in Obese Mice. J. Pharm. Pharmacogn. Res..

[35] Noguera-Navarro C., Montoro-García S., Orenes-Piñero E. (2023). Hydroxytyrosol: Its Role in the Prevention of Cardiovascular Diseases. Heliyon.

[36] Cornali K., Di Lauro M., Marrone G., Masci C., Montalto G., Giovannelli A., Schievano C., Tesauro M., Pieri M., Bernardini S. (2025). The Effects of a Food Supplement, Based on Co-Micronized Palmitoylethanolamide (PEA)–Rutin and Hydroxytyrosol, in Metabolic Syndrome Patients: Preliminary Results. Nutrients.

[37] Binou P., Stergiou A., Kosta O., Tentolouris N., Karathanos V.T. (2023). Positive Contribution of Hydroxytyrosol-Enriched Wheat Bread to HbA1c Levels, Lipid Profile, Markers of Inflammation and Body Weight in Subjects with Overweight/Obesity and Type 2 Diabetes Mellitus. Eur. J. Nutr..

[38] Khound P., Gurumayum N., Devi R. (2025). Amelioration of Atherosclerotic Complications and Dyslipidemia by Verbascoside-Enriched Fraction of *Clerodendrum glandulosum* Leaves Targeting LDL-R and LXR-Mediated Reverse Cholesterol Transport. Chin. Herb. Med..

[39] Chen S., Liu H., Wang S., Jiang H., Gao L., Wang L., Teng L., Wang C., Wang D. (2022). The Neuroprotection of Verbascoside in Alzheimer’s Disease Mediated through Mitigation of Neuroinflammation via Blocking NF-κB-P65 Signaling. Nutrients.

[40] Moris J.M., Heinold C., Blades A., Koh Y. (2022). Nutrient-Based Appetite Regulation. J. Obes. Metab. Syndr..

[41] Rahemi M., Alizadeh M. (2018). The Association of Energy Intake and Expenditure, Macronutrients, Glycemic Index and Load, and General Characteristics with Postprandial Peptide YY 3-36 Serum Levels. Crescent J. Med. Biol. Sci..

[42] Li R., Zhu Q., Wang X., Wang H. (2022). Mulberry Leaf Polyphenols Alleviated High-Fat Diet-Induced Obesity in Mice. Front. Nutr..

[43] Pacifici F., Malatesta G., Mammi C., Pastore D., Marzolla V., Ricordi C., Chiereghin F., Infante M., Donadel G., Curcio F. (2023). A Novel Mix of Polyphenols and Micronutrients Reduces Adipogenesis and Promotes White Adipose Tissue Browning via UCP1 Expression and AMPK Activation. Cells.

[44] Shahidi F., Danielski R. (2024). Review on the Role of Polyphenols in Preventing and Treating Type 2 Diabetes: Evidence from In Vitro and In Vivo Studies. Nutrients.

[45] Delgadillo-Puga C., Torre-Villalvazo I., Noriega L.G., Rodríguez-López L.A., Alemán G., Torre-Anaya E.A., Cariño-Cervantes Y.Y., Palacios-Gonzalez B., Furuzawa-Carballeda J., Tovar A.R. (2023). Pecans and Its Polyphenols Prevent Obesity, Hepatic Steatosis and Diabetes by Reducing Dysbiosis, Inflammation, and Increasing Energy Expenditure in Mice Fed a High-Fat Diet. Nutrients.

[46] Langhi C., Vallier M., Bron A., Otero Y.F., Maura M., Le Joubioux F., Blomberg N., Giera M., Guigas B., Maugard T. (2024). A Polyphenol-Rich Plant Extract Prevents Hypercholesterolemia and Modulates Gut Microbiota in Western Diet-Fed Mice. Front. Cardiovasc. Med..

[47] Dzah C.S., Asante-Donyinah D., Letsyo E., Dzikunoo J., Adams Z.S. (2023). Dietary Polyphenols and Obesity: A Review of Polyphenol Effects on Lipid and Glucose Metabolism, Mitochondrial Homeostasis, and Starch Digestibility and Absorption. Plant Foods Hum. Nutr..

[48] Zhao H., Shen C., Wu Z., Zhang Z., Xu C. (2020). Comparison of Wheat, Soybean, Rice, and Pea Protein Properties for Effective Applications in Food Products. J. Food Biochem..

[49] Tomšik A., Pavlić B., Vladić J., Cindrić M., Jovanov P., Sakač M., Mandić A., Vidović S. (2017). Subcritical Water Extraction of Wild Garlic (*Allium Ursinum* L.) and Process Optimization by Response Surface Methodology. J. Supercrit. Fluids.

[50] Zeković Z., Vidović S., Vladić J., Radosavljević R., Cvejin A., Elgndi M.A., Pavlić B. (2014). Optimization of Subcritical Water Extraction of Antioxidants from Coriandrum Sativum Seeds by Response Surface Methodology. J. Supercrit. Fluids.

[51] Ahmadian-Kouchaksaraie Z., Niazmand R., Najafi M.N. (2016). Optimization of the Subcritical Water Extraction of Phenolic Antioxidants from *Crocus sativus* Petals of Saffron Industry Residues: Box-Behnken Design and Principal Component Analysis. Innov. Food Sci. Emerg. Technol..

[52] López-Bascón M.A., De Castro M.L. (2020). Soxhlet Extraction. Liquid-Phase Extraction.

[53] Zhao H., Avena-Bustillos R.J., Wang S.C. (2022). Extraction, Purification and In Vitro Antioxidant Activity Evaluation of Phenolic Compounds in California Olive Pomace. Foods.

[54] McCleary B.V. (2023). Measurement of Dietary Fiber: Which AOAC Official Method of AnalysisSM to Use. J. AOAC Int..

[55] Zhao H., Kim Y., Avena-Bustillos R.J., Nitin N., Wang S.C. (2023). Characterization of California Olive Pomace Fractions and Their in Vitro Antioxidant and Antimicrobial Activities. LWT-Food Sci. Technol..

[56] Chehade S.B., Green G.B.H., Graham C.D., Chakraborti A., Vashai B., Moon A., Williams M.B., Vickers B., Berryhill T., Van Der Pol W. (2022). A Modified Standard American Diet Induces Physiological Parameters Associated with Metabolic Syndrome in C57BL/6J Mice. Front. Nutr..

[57] Haj F.G. (2019). UC Davis-Intraperitoneal Glucose Tolerance Test. protocols.io.

[58] Nagy C., Einwallner E. (2018). Study of In Vivo Glucose Metabolism in High-Fat Diet-Fed Mice Using Oral Glucose Tolerance Test (OGTT) and Insulin Tolerance Test (ITT). J. Vis. Exp..

[59] Acosta-Cota S.D.J., Aguilar-Medina E.M., Ramos-Payán R., Ruiz-Quiñónez A.K., Romero-Quintana J.G., Montes-Avila J., Rendón-Maldonado J.G., Sánchez-López A., Centurión D., Osuna-Martínez U. (2019). Histopathological and Biochemical Changes in the Development of Nonalcoholic Fatty Liver Disease Induced by High-Sucrose Diet at Different Times. Can. J. Physiol. Pharmacol..

